# Impact of CFTR modulators on caregiver burden in cystic fibrosis: a cross-sectional study in Brazil

**DOI:** 10.1186/s13023-026-04514-z

**Published:** 2026-07-23

**Authors:** Verônica Del Gragnano Stasiak Bednarczuk Oliveira, Vinícius Bednarczuk Oliveira, Marise Basso Amaral, Marilis Dallarmi Miguel

**Affiliations:** 1 Unidos pela Vida - Brazilian Institute of Cystic Fibrosis Care, Supera Consulting and Federal University of Paraná (UFPR), Curitiba, Brazil; 2 Unidos pela Vida - Brazilian Institute of Cystic Fibrosis Care and Supera Consulting, Curitiba, Brazil; 3 Unidos pela Vida - Brazilian Institute of Cystic Fibrosis Care and Federal University Fluminense, Rio de Janeiro, Brazil; 4 Postgraduate Program in Pharmaceutical Sciences, Department of Pharmacy, Federal University of Paraná (UFPR), Curitiba, Paraná Brazil

**Keywords:** Cystic fibrosis, Caregiver burden, Quality of life, CFTR protein

## Abstract

**Objective:**

To assess caregiver burden, health-related quality of life, and work productivity among primary caregivers of individuals with cystic fibrosis (CF) in Brazil, comparing caregivers of patients using elexacaftor/tezacaftor/ivacaftor (ETI) with those of non-users, and analyzing differences according to the age group of the care recipient.

**Methods:**

This cross-sectional study was conducted between September and November 2025 using an online questionnaire. Eligible participants were primary caregivers of individuals with CF living in Brazil. Collected data included sociodemographics, CFTR modulator use, caregiving time, and out-of-pocket health-related expenses. Validated instruments included the 12-item Zarit Burden Interview (ZBI-12), the EQ-5D-3L (utility index and Visual Analogue Scale), and the Work Productivity and Activity Impairment Questionnaire for Caregivers (WPAI: CG). Descriptive analyses and group comparisons were stratified by ETI use and age group of the care recipient.

**Results:**

A total of 174 caregivers from all Brazilian regions participated; 90.2% were mothers, with a median age of 35 years (IQR: 28–45). The individuals receiving care had a median age of 11 years (IQR: 7–14) among modulator users and 3 years (IQR: 1–7) among non-users, and 44.8% used CFTR modulators. Caregivers of non-modulator users dedicated significantly more time to daily caregiving (300 min [IQR: 180–300] vs. 180 min [IQR: 90–240]; *p* = 0.002) and reported more frequent consultations (*p* = 0.003). ZBI-12 scores were higher in the no-modulator group (20.0 [IQR: 16.0–25.2] vs. 15.5 [IQR: 11.2–23.0]; *p* = 0.006), with the greatest difference observed among caregivers of children under 5 years (20.0 vs. 14.0; *p* = 0.003), a group currently without access to modulators through the public health system. EQ-VAS was lower in the no-modulator group (65.0 [IQR: 50.0–80.0] vs. 70.0 [IQR: 50.0–80.0]; *p* = 0.041). Absenteeism (15.8% [IQR: 0.0–28.6] vs. 0.0% [IQR: 0.0–16.4]; *p* = 0.034), presenteeism (50.0% [IQR: 5.0–60.0] vs. 20.0% [IQR: 0.0–50.0]; *p* = 0.048), and activity impairment (50.0% [IQR: 22.5–70.0] vs. 30.0% [IQR: 0.0–60.0]; *p* = 0.011) were significantly higher among caregivers without modulator access.

**Conclusion:**

Use of CFTR modulators was associated with lower caregiver burden, better health perception, and reduced work productivity loss.

## Introduction

Cystic fibrosis (CF) is a chronic, progressive, autosomal recessive genetic disorder caused by mutations in the *CFTR (Cystic Fibrosis Transmembrane Conductance Regulator)* gene, which encodes the CFTR protein, primarily affecting the respiratory and digestive systems [1, 2]. Its pathophysiology is associated with the abnormal production of thick and viscous secretions, leading to recurrent pulmonary infections, pancreatic insufficiency, and malnutrition [1, 3, 4].

Although classified as a rare disease, CF is among the most common genetic disorders in the Caucasian population [5, 6]. In Brazil, the estimated incidence ranges from 1 in every 7,576 to 8,400 live births, with significant regional variation [7, 8]. According to the 2023 Brazilian Cystic Fibrosis Registry (REBRAFC) [9], there are 5,930 registered patients currently in follow-up across the country. Despite this, challenges persist regarding early diagnosis, accurate case reporting, and access to specialized treatments.

Managing CF requires intensive and continuous care, involving multiple daily interventions such as nebulization, respiratory physiotherapy, pancreatic enzyme replacement, antibiotics, and CFTR modulator therapies, available only for specific mutations [10–12]. This complex care regimen imposes a substantial burden on caregivers, particularly in households with young children, where caregiving is often the responsibility of a single family member, typically a woman [13, 14].

CFTR modulator therapies have revolutionized the treatment of CF by acting directly on the underlying molecular defect of the disease [15–18]. These therapies lead to significant clinical improvements in eligible patients, with evidence of gains in pulmonary function, nutritional status, and quality of life, as well as reduced frequency of pulmonary exacerbations [16, 17, 19]. Although their clinical impact is well established, access remains unequal in Brazil, as in many other countries, particularly for children. This disparity may influence not only clinical outcomes but also the level of burden experienced by caregivers.

In Brazil [20], two CFTR modulators are currently incorporated into the Unified Health System (SUS). Ivacaftor (Kalydeco^®^) was incorporated in December 2020 and is indicated for patients aged six years or older carrying at least one gating mutation (G551D, G1244E, G1349D, G178R, G551S, S1251N, S1255P, S549N, or S549R), as well as for patients aged 18 years or older with one copy of the R117H mutation. The combination therapy elexacaftor/tezacaftor/ivacaftor (ETI; Trikafta^®^) was incorporated in September 2023 and is indicated for patients aged six years or older with at least one copy of the F508del mutation. Other CFTR modulators have been submitted for evaluation but were not incorporated into the SUS.

Despite the relevance of the topic, few studies in Brazil have quantitatively assessed the burden of care from the perspective of informal caregivers, such as family members or unpaid individuals responsible for the daily care of people with CF. Furthermore, little is known about how the introduction of CFTR modulators may affect caregivers’ perceived burden and quality of life. In this context, the present study aimed to assess the burden of care, quality of life, and productivity-related impacts among primary caregivers of individuals with CF in Brazil, comparing caregivers of ETI users with those of non-users.

## Methods

This is a cross-sectional study based on primary data collected from caregivers of individuals with CF in Brazil. Data collection was conducted between September and November 2025 through an online questionnaire hosted on the Google Forms platform. Eligible participants were caregivers aged 18 years or older serving as the primary caregiver of an individual with a confirmed diagnosis of CF, regardless of patient age. For the purposes of this study, CFTR modulator use was defined as the use of ETI, as this was the only modulator therapy reported by participants in the sample. Dissemination was carried out by Instituto Unidos pela Vida, a nonprofit organization dedicated to CF, through its institutional communication channels, social media, and partner support associations, with the goal of reaching caregivers across all regions of Brazil. After accepting the online Informed Consent Form (ICF), participants were directed to the screening questions and thematic sections of the survey.

The questionnaire consisted of three main sections: (1) sociodemographic characteristics; (2) validated instruments, including the 12-item Zarit Burden Interview (ZBI-12), the Work Productivity and Activity Impairment Questionnaire – Caregiver Version (WPAI: CG), and the EQ-5D-3 L with Visual Analogue Scale (VAS); and (3) care logistics, including frequency of medical appointments, travel time, daily caregiving hours, and out-of-pocket expenses for medications or procedures not covered by the SUS.

### Data analysis

Data were organized in electronic spreadsheets and analyzed using Microsoft Excel^®^, Python (Google Colab platform) with the pandas and scipy.stats libraries, and R with the eq5d package for calculation of EQ-5D-3L utility index scores. The Shapiro-Wilk test was applied to assess the normality of continuous variables. Based on the results, categorical variables were described as absolute and relative frequencies [n (%)], and continuous variables as median with interquartile range (IQR: P25–P75). For results presented in U.S. dollars (USD), the average exchange rate for the six-month period preceding data collection (June to November 2025) was applied, corresponding to BRL 5.47 per USD.

Scores from the standardized instruments were processed according to the methodological guidelines of each scale. The EQ-5D-3L utility index was calculated using Brazilian valuation weights, and the Visual Analogue Scale (VAS) was analyzed as a continuous variable. WPAI: CG domain scores were converted into percentages using the formulas provided in the original instrument.

To compare scores obtained from the standardized instruments and care-related variables between caregivers of patients with and without CFTR modulator use, the Mann-Whitney U test was applied, given the non-normal distribution observed across most variables. Although the ZBI-12 presented normal distribution in both groups, the Mann-Whitney U test was maintained for all comparisons to ensure analytical consistency. The level of statistical significance was set at 5% (*p* < 0.05).

### Ethical considerations

The study was approved by a Research Ethics Committee of the International University Center UNINTER (Approval No. 7.879.811) and conducted in accordance with national and international ethical standards for research involving human subjects. All participants provided informed consent, and both respondent anonymity and data confidentiality were strictly maintained.

## Results

A total of 174 primary caregivers of individuals with CF participated in the study and provided informed consent. Among them, 78 (44.8%) were caregivers of patients receiving CFTR modulator therapy, whereas 96 (55.2%) cared for patients not using modulators. All participants completed the ZBI-12 and EQ-5D-3L questionnaires. The WPAI: CG was completed only by caregivers who were employed at the time of the survey (*n* = 96).

The sociodemographic data of these caregivers are presented in Table 1. Most participants were mothers (90.2%), residing across all regions of Brazil, with a higher concentration in the Southeast region. The majority of caregivers reported being married and had completed either high school or higher education. Regarding the individuals under their care, most were female (54.0%), with a median age of 11 years (IQR: 7–14) among modulator users and 3 years (IQR: 1–7) among non-users.

**Table 1:** Sociodemographic characteristics of caregivers and patients

Caregivers		
**Relationship**		**n (%)**
Mother		157 (90.2%)
Father		17 (9.8%)
**Age**		**Median (IQR)**
Age (years)		35 (28–45)
**Marital status**		**n (%)**
Married		95 (54.6%)
Single		30 (17.2%)
Other		49 (28.2%)
**Education level**		**n (%)**
Completed high school		57 (32.8%)
Completed postgraduate		38 (21.8%)
Other		79 (45.4%)
**Monthly family income**		**Median (IQR)**
Monthly family income (USD)		645 (194-1,033)
**Region**		**n (%)**
North		9 (5.2%)
Northeast		40 (23.0%)
Central-West		5 (2.9%)
Southeast		69 (39.6%)
South		51 (29.3%)
**Patients**		**n (%)**
**Use of CFTR modulators**		78 (44.8%)
**Sex**		**n (%)**
Female		94 (54.0%)
Male		80 (46.0%)
**Age (Years)**		**Median (IQR)**
Modulator use		11 (7–14)
No modulator use		3 (1–7)
**Access to CFTR modulators**		**n (%)**
SUS		47 (60.3%)
Judicial action		24 (30.8%)
Out-of-pocket / crowdfunding / donations		7 (8.9%)

As shown in Table 2, caregivers of patients not using CFTR modulators dedicated significantly more time to daily caregiving (300 min [IQR: 180–300] vs. 180 min [IQR: 90–240]; *p* = 0.002) and reported more frequent medical consultations (*p* = 0.003). No significant differences were observed between groups in distance to the referral center, travel time, time at appointment, or physiotherapy-related variables. Among caregivers who reported out-of-pocket expenses on non-SUS medications, physiotherapy, or psychological support, median monthly costs were USD 37 (IQR: 37–146) in the modulator group and USD 82 (IQR: 37–98) in the no-modulator group, representing a relevant additional financial burden on families already facing the daily demands of caring for a person with CF.

**Table 2:** Care-related burden and out-of-pocket costs among caregivers of individuals with cystic fibrosis according to CFTR modulator use

Variable	Modulator use (*n* = 78)	No modulator use (*n* = 96)	*p*-value
Daily caregiving (min)	180 (90–240)	300 (180–300)	0.002
Consultation frequency (days)	90 (60–90)	90 (30–90)	0.003
Distance to referral center (km)	150 (23–250)	75 (18–250)	0.741
Travel time to center (min)	90 (45–180)	90 (45–210)	0.822
Time at appointment (min)	180 (180–300)	180 (180–300)	0.825
Monthly expense on non-SUS medications (USD)	37 (37–146)	82 (37–98)	0.915
Physiotherapy frequency (times/week)	3 (2–6)	5 (2–7)	0.331
Physiotherapy session duration (min)	25 (25–45)	25 (25–45)	0.487
Monthly physiotherapy cost (USD)	82 (37–146)	82 (82–146)	0.697
Monthly psychology cost (USD)	82 (37–98)	82 (82–229)	0.405

Note: Values expressed as median (IQR: P25–P75). p-values derived from Mann-Whitney U test. Monthly expense on non-SUS medications (*n* = 56/72), physiotherapy frequency (*n* = 58/72), physiotherapy duration (*n* = 59/71), physiotherapy cost (*n* = 15/17), and psychology cost (*n* = 12/5) were restricted to caregivers reporting the respective expense or service

As shown in Table 3, caregivers of patients not using CFTR modulators reported significantly higher burden across most dimensions assessed. Caregiver burden was significantly higher in the no-modulator group (ZBI-12: 20.0 [IQR: 16.0–25.2] vs. 15.5 [IQR: 11.2–23.0]; *p* = 0.006), as was overall health perception (EQ-VAS: 65.0 [IQR: 50.0–80.0] vs. 70.0 [IQR: 50.0–80.0]; *p* = 0.041). The EQ-5D-3L utility index did not differ significantly between groups (*p* = 0.371), possibly reflecting the multidimensional nature of the instrument, which aggregates mobility, self-care, usual activities, pain/discomfort, and anxiety/depression into a single score.

**Table 3:** Caregiver burden, health-related quality of life, and work productivity according to CFTR modulator use

Instrument	Modulator use (*n* = 78)	No modulator use (*n* = 96)	*p*-value
ZBI-12	15.5 (11.2–23.0)	20.0 (16.0–25.2)	0.006
EQ-5D-3L Index	0.875 (0.806–0.929)	0.873 (0.750–0.929)	0.371
EQ-VAS	70.0 (50.0–80.0)	65.0 (50.0–80.0)	0.041
WPAI Absenteeism (%)	0.0 (0.0–16.4)	15.8 (0.0–28.6)	0.034
WPAI Presenteeism (%)	20.0 (0.0–50.0)	50.0 (5.0–60.0)	0.048
WPAI Overall Work Impairment (%)	20.0 (0.0–60.9)	59.7 (5.0–74.4)	0.055
WPAI Activity Impairment (%)	30.0 (0.0–60.0)	50.0 (22.5–70.0)	0.011

Note: Values expressed as median (IQR: P25–P75). p-values derived from Mann-Whitney U test. WPAI domains were calculated exclusively for employed caregivers (Modulator use: *n* = 46; No modulator use: *n* = 50)

Among the 96 employed caregivers, including 81 mothers (51.6% of responding mothers) and 15 fathers (88.2% of responding fathers), the impact on work productivity was substantial and consistent across domains. Absenteeism, presenteeism, and activity impairment were all significantly higher among caregivers of patients without modulator access (*p* = 0.034, *p* = 0.048, and *p* = 0.011, respectively). Overall work impairment showed a clinically meaningful difference that did not reach statistical significance (*p* = 0.055), potentially reflecting limited statistical power due to sample size.

**Fig. 1:**
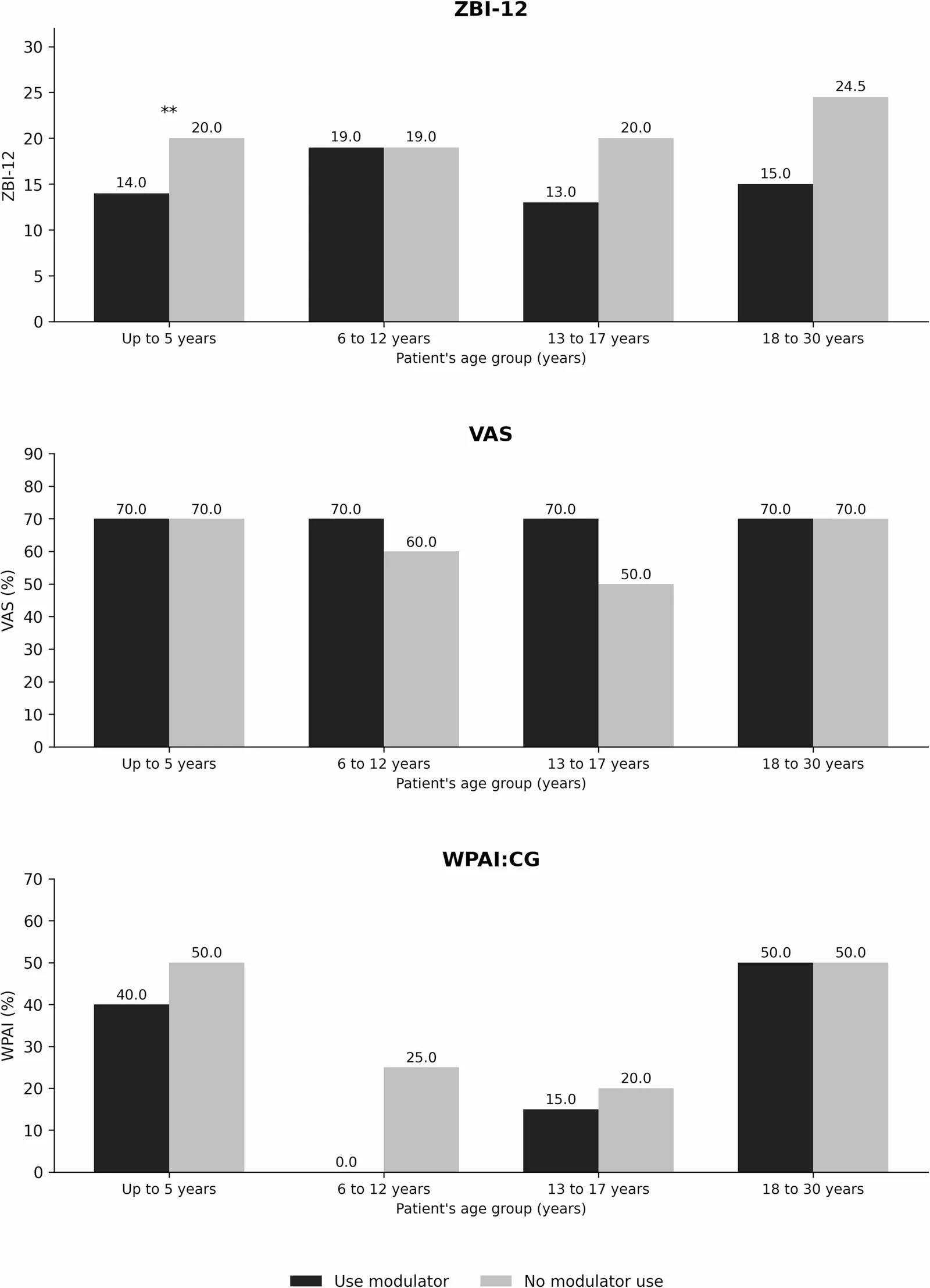
Comparison of median scores for caregiver burden (ZBI-12), health perception (VAS), and work-related activity impairment (WPAI: CG) among caregivers of individuals with and without CFTR modulator use, according to the patient’s age group. Note: ZBI-12: 12-item Zarit Burden Interview; VAS: Visual Analogue Scale (EQ-5D-3L); WPAI: CG: Work Productivity and Activity Impairment Questionnaire – Caregiver Version. Values represent medians. ** p < 0.01 (Mann-Whitney U test). WPAI: CG statistical comparison was not performed for the 13–17 and 18–30 age groups due to insufficient sample size

As illustrated in Fig. 1, caregivers of patients using CFTR modulators reported lower burden, better health perception, and less work-related impairment across nearly all age groups compared to caregivers of patients not using modulators. The most pronounced and statistically significant difference was observed among caregivers of children under five years of age (ZBI-12: 14.0 vs. 20.0; *p* = 0.003), a group currently without access to CFTR modulator therapy through the SUS, highlighting the potential burden reduction that could be achieved with broader treatment access.

## Discussion

The findings of this study demonstrate that caregivers of individuals with CF not using ETI reported significantly higher caregiver burden, worse health perception, greater work productivity loss, and more time dedicated to daily caregiving compared to caregivers of ETI users. These differences were particularly pronounced among caregivers of children under five years of age, a group currently excluded from ETI access through the SUS.

The daily management of the disease, which includes multiple clinical interventions, respiratory therapies, nutritional monitoring, and continuous follow-up, directly interferes with the organization of personal and professional routines. These results are consistent with previous studies showing high levels of burden among caregivers of individuals with chronic and rare diseases [21–23], including CF [24, 25].

The sample was predominantly composed of mothers, reinforcing the central role of women as caregivers in the context of chronic illness, a phenomenon widely described in the literature [26–28]. Most participants were of working age, highlighting the overlap between caregiving responsibilities and formal employment demands. More than half of the mothers interviewed were employed, underscoring the dual burden of caregiving and professional roles.

The regional distribution of participants partially reflected the geographic distribution of CF patients registered in Brazil (REBRAFC) [9], with predominance in the Southeast (39.6% vs. 47.7%) and South (29.3% vs. 22.9%) regions, which together accounted for approximately 68% of the sample and 70% of nationally registered cases. The Northeast was slightly overrepresented (23.0% vs. 16.5%), while the Central-West was underrepresented (2.9% vs. 6.4%), likely reflecting limitations inherent to convenience sampling via digital dissemination channels. Nevertheless, the presence of participants from all five Brazilian regions strengthens the national scope of the findings.

The age difference between groups, with a median of 11 years (IQR: 7–14) among modulator users versus 3 years (IQR: 1–7) among non-users, directly reflects the current SUS eligibility criteria [20], which restrict ETI access to patients aged six years or older carrying at least one F508del mutation, leaving younger children and those with non-responsive mutations without access to disease-modifying therapy and whose caregivers, as shown in our data, bear the highest burden [20, 29].

Notably, a substantial proportion of patients were aged five years or younger, a group currently excluded from ETI access through the SUS, with only a minority obtaining therapy exclusively through legal action. These data highlight a significant gap in early access to treatment during a critical phase of disease development.

Caregivers of patients not using modulators dedicated significantly more time to daily caregiving (*p* = 0.002), with the total time commitment on appointment days potentially reaching up to 11 h when combining caregiving, travel, and time at the referral center. This reflects the complexity of care provided by families, especially in cases involving multiple comorbidities or logistical barriers [13, 30, 31].

The financial burden was also considerable, with caregivers of patients not using modulators reporting relevant out-of-pocket expenses across medications, physiotherapy, and psychological support not covered by the SUS. These findings are consistent with the literature, which reports that indirect costs can represent up to one-third of household income in countries with partial or unequal public coverage, reinforcing the need for comprehensive public policies to support caregivers and reduce financial barriers to home-based care [25, 32, 33].

From a psychosocial perspective, caregiver burden (ZBI-12) was significantly lower among caregivers of patients using ETI (*p* = 0.006), with scores consistent with the established cutoff of 13 points for burden detection. Health perception (EQ-VAS) also differed significantly between groups (*p* = 0.041), suggesting better overall wellbeing among caregivers in the modulator group. The EQ-5D-3L utility index did not differ significantly (*p* = 0.371), possibly reflecting the multidimensional structure of the instrument, which may not fully capture the subjective dimensions of caregiver wellbeing.

Together, these findings suggest that the incorporation of disease-modifying therapies may have beneficial indirect effects not only for patients but also for caregivers. Although international literature already reports reductions in hospitalizations and symptoms with modulator use [17, 19, 34], studies exploring caregiver-specific outcomes remain scarce.

Regarding work productivity, impairment was substantially and consistently higher among caregivers of patients not using modulators across all WPAI: CG domains, with significant differences in presenteeism and activity impairment (*p* = 0.048 and *p* = 0.011, respectively). These findings highlight the indirect effects of CF on caregiver functionality and autonomy, particularly among women, who represented the majority of participants and showed lower employment rates compared to fathers, a pattern previously noted in other studies [23, 27, 35, 36]. Even when caregivers maintained employment, the impacts on productivity and daily life were substantial.

Age-stratified analysis (Fig. 1) revealed particularly pronounced differences among caregivers of children aged five years or younger, a group currently without access to CFTR modulator therapy through the SUS. These caregivers reported the highest caregiver burden (*p* = 0.003) and consistently lower health perception across all domains assessed, reinforcing the urgent need to expand modulator access to younger age groups.

These findings highlight a consistent pattern of better outcomes among caregivers of individuals using modulators, including reduced burden, better health perception, and less professional impact. Since CFTR modulators are not yet available through the SUS for children under six years of age, these families face a greater care burden without access to the benefits of these therapies, amplifying the personal, and professional strain on caregivers.

Studies from various contexts reinforce that the psychosocial burden associated with caring for individuals with CF is multifactorial and exacerbated by disease complexity, particularly during phases of greater functional dependency [30, 37]. Previous research has identified high levels of stress, anxiety, and emotional strain among caregivers, especially mothers, who often face dual roles and challenges in balancing caregiving with other responsibilities [21, 25, 26, 28].

The reduction in symptom burden and hospitalization needs associated with CFTR modulators may help alleviate the emotional and physical strain on caregivers, even though these effects are not always directly measured [19, 35]. The literature still lacks robust longitudinal studies evaluating the indirect effects of disease-modifying therapies on caregivers’ mental health, quality of life, and functional capacity, especially in countries with universal public health systems such as Brazil.

This study has some limitations. The sample was non-probabilistic and collected through an online form, which may have introduced selection bias by favoring participation from caregivers with greater engagement and internet access. Additionally, the cross-sectional design prevents causal inference and does not capture changes over time. All data were self-reported, which may introduce recall or social desirability bias. Nevertheless, internationally validated instruments were used (ZBI-12, EQ-5D-3L, WPAI: CG), with good acceptability in caregiver research.

Future longitudinal studies with more robust sampling strategies are needed to better understand the caregiving trajectory and the economic and psychosocial impacts over the course of disease progression.

In the Brazilian context, it is important to highlight that most CF care is provided through the SUS, a universal public system offering free access to various therapies and procedures. As shown in this study, the SUS has been the main provider of high-cost treatments, such as CFTR modulators, although access inequities persist, particularly among patients with rare mutations or those in age groups not yet covered by incorporation protocols.

Nevertheless, the results should be interpreted in light of the specificities of the Brazilian healthcare system, which is marked by regional inequalities, heterogeneity in the availability of specialized services, and structural limitations that affect the organization of care. These factors may significantly influence both the caregiver’s journey and the effectiveness of the care provided. Therefore, the generalization of the findings to other countries or healthcare systems should take into account differences in public policies, institutional support, and care delivery models.

## Conclusion

This study demonstrated that caring for individuals with CF in Brazil places a substantial burden on caregivers, particularly mothers of young children, with significant repercussions on health perception, work productivity, and family finances. The complex daily care routine, frequent travel to referral centers, and out-of-pocket expenses not covered by the SUS contribute to amplifying this burden. Importantly, the use of CFTR modulators was associated with lower caregiver burden and better health perception, suggesting indirect benefits of disease-modifying therapies for family dynamics and caregiver autonomy.

These findings highlight the urgent need to expand equitable access to modulators, especially for children under six years of age and patients with mutations not yet covered by public policies. Psychosocial support strategies, flexible work policies, and social protection measures that recognize the impact of chronic caregiving on families are equally necessary. Ensuring continuity and expansion of SUS coverage, with a focus on comprehensive care and reduction of regional inequalities, remains essential.

Future longitudinal studies may clarify how caregiving burden evolves over time and allow more precise assessment of the effects of emerging therapies on caregiver outcomes. The data presented here represent a novel contribution in the Brazilian context and may inform health technology assessment decisions, guide public policy planning, and support the strengthening of caregiver support networks in CF.

## Data Availability

Due to privacy protections, the datasets generated during and analyzed during the current study are not publicly available. The data supporting our findings are available upon reasonable request through the corresponding author, Verônica Oliveira, via veronica.bednarczuk@gmail.com.

## Abbreviations

CF: Cystic Fibrosis
CFTR: Cystic Fibrosis Transmembrane Conductance Regulator
EQ-5D-3L: EuroQoL 5-Dimension 3-Level
ETI: Elexacaftor/tezacaftor/ivacaftor
IQR: Interquartile Range
REBRAFC: Brazilian Cystic Fibrosis Registry
SUS: Unified Health System
VAS: Visual analogue scale
WPAI:CG: Work Productivity and Activity Impairment questionnaire as adapted for caregiving
ZBI-12: Zarit Burden Interview – 12 items
